# Glucocorticoid Receptor Gene Polymorphisms and Femoral Head Osteonecrosis

**DOI:** 10.3390/medicina62061068

**Published:** 2026-05-31

**Authors:** De-Yi Liu, I-Chang Lai, Nien-En Ku, Chang-Yuan Hsu, Chu-Ching Wang, Shih-Chan Hsu, Jih-Yang Ko, Shu-Jui Kuo

**Affiliations:** 1 Department of Education, China Medical University Hospital, Taichung 404327, Taiwan; deyil845@gmail.com (D.-Y.L.); lai.i.chang.58@gmail.com (I.-C.L.); nicheerrr1007@gmail.com (N.-E.K.); mimiman881106@gmail.com (C.-Y.H.); abc20000606@gmail.com (C.-C.W.); 2 School of Medicine, China Medical University, Taichung 404328, Taiwan; jamieee0424@gmail.com; 3 Department of Orthopedic Surgery, Kaohsiung Chang Gung Memorial Hospital, Kaohsiung 833401, Taiwan; kojy@cgmh.org.tw; 4 Department of Orthopedic Surgery, China Medical University Hospital, Taichung 404327, Taiwan

**Keywords:** genetic polymorphisms, *NR3C1*, osteonecrosis of the femoral head, single-nucleotide polymorphisms

## Abstract

*Background and Objectives*: Non-traumatic osteonecrosis of the femoral head (ONFH) is a multifactorial disorder influenced by both environmental and genetic factors. The nuclear receptor subfamily 3 group C member 1 (*NR3C1*) gene encodes the glucocorticoid receptor, which plays a key role in bone metabolism, vascular regulation, and stress response. This study aimed to investigate the association between *NR3C1* polymorphisms and susceptibility to ONFH, with particular emphasis on age-related genetic effects. *Materials and Methods*: A hospital-based case–control study was conducted using genotyping data from the Taiwan Biobank version 2 (TWBv2) custom array. A total of 609 patients with ONFH and 2436 age- and sex-matched controls were included. Forty-nine single-nucleotide polymorphisms (SNPs) within or near the *NR3C1* gene with a minor allele frequency greater than 5% were analyzed. Logistic regression models were applied to estimate odds ratios (ORs) and 95% confidence intervals (CIs) under multiple genetic inheritance models. Age-stratified analyses were also performed. *Results*: Among the analyzed SNPs, rs28593206 and rs315199 demonstrated nominally significant differences in allele distributions between cases and controls (*p* = 0.024 and *p* = 0.009, respectively), with minor alleles showing nominal associations with increased odds of ONFH. Additional exploratory analyses under different genetic models identified several SNPs with nominal associations with ONFH susceptibility, while rs7709864 showed a possible nominal protective association. In age-stratified analyses, several SNPs showed nominal associations with ONFH risk among individuals older than 40 years, whereas no nominally significant associations were observed in younger individuals. *Conclusions*: This exploratory study suggests that *NR3C1* polymorphisms may be nominally associated with susceptibility to ONFH, particularly among individuals older than 40 years. However, because no formal correction for multiple comparisons was performed and detailed etiological data were unavailable, these findings should be interpreted cautiously and regarded as hypothesis-generating rather than clinically applicable evidence. Further studies incorporating larger cohorts, etiological stratification, multiple-testing correction, replication analyses, and functional validation are required to confirm these observations and clarify the underlying biological mechanisms.

## 1. Introduction

Non-traumatic osteonecrosis of the femoral head (ONFH) is a progressive and debilitating orthopedic disorder characterized by bone necrosis secondary to impaired blood supply in the absence of direct trauma [1,2]. ONFH commonly affects relatively young and active individuals and frequently progresses to femoral head collapse, ultimately necessitating total hip arthroplasty [3]. Because many patients require surgical intervention at a comparatively early age, the long-term durability and complications of hip arthroplasty remain important clinical concerns. Historically, some patients underwent metal-on-metal hip arthroplasty, which has been associated with local adverse tissue reactions and systemic complications related to metal ion release and metal debris [4]. These issues further highlight the substantial clinical burden of ONFH and underscore the importance of improving early disease identification and risk stratification.

Despite extensive research, the pathogenesis of ONFH is complex and multifactorial, involving the interplay of environmental exposures and genetic susceptibility. Established risk factors—including corticosteroid use, excessive alcohol consumption, smoking, and systemic diseases—account for the majority of cases; however, only a subset of exposed individuals develops ONFH, suggesting an important role for genetic predisposition [5,6].

Single-nucleotide polymorphisms (SNPs) represent the most common form of genetic variation and may influence gene expression, protein function, and downstream biological pathways [7,8,9,10]. Increasing evidence indicates that genetic factors contribute to ONFH susceptibility through mechanisms related to vascular dysfunction, lipid metabolism, inflammation, and impaired bone remodeling [5]. While genome-wide association studies (GWAS) have identified multiple loci associated with complex diseases, their findings often explain only a limited proportion of heritability and may lack clear biological interpretability. Consequently, hypothesis-driven candidate gene approaches remain valuable, particularly when grounded in well-established disease mechanisms, as they allow for focused investigation of biologically relevant pathways and facilitate mechanistic interpretation [11,12,13,14].

**Figure 1:**
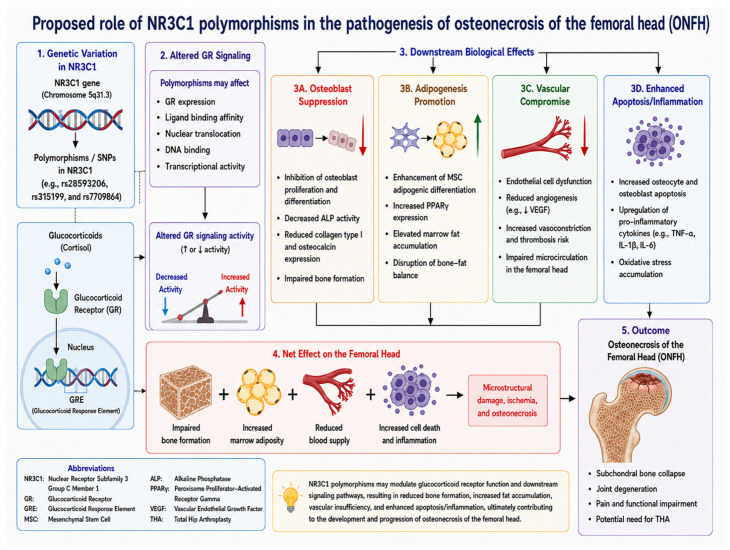
Proposed role of *NR3C1* polymorphisms in the pathogenesis of osteonecrosis of the femoral head (ONFH). Genetic polymorphisms within the *NR3C1* gene may alter glucocorticoid receptor (GR) signaling through effects on receptor expression, ligand binding affinity, nuclear translocation, DNA binding, or transcriptional activity. Altered GR signaling may subsequently contribute to multiple downstream biological processes implicated in ONFH development, including suppression of osteoblast proliferation and differentiation, promotion of adipogenesis, vascular dysfunction, and enhanced apoptosis and inflammation. These pathological changes may collectively impair bone remodeling, reduce blood supply to the femoral head, and promote microstructural damage and osteonecrosis. Black arrows indicate the proposed mechanistic sequence from NR3C1 polymorphisms to altered glucocorticoid receptor signaling, downstream biological effects, and ONFH development. Upward arrows denote increased activity or promotion, whereas downward arrows denote decreased activity or suppression. The schematic illustrates a biologically plausible mechanism linking *NR3C1* polymorphisms to increased susceptibility to ONFH.

The nuclear receptor subfamily 3, group C, member 1 (*NR3C1*) gene encodes the glucocorticoid receptor (GR), a key regulator of glucocorticoid signaling [15,16]. Activation of GR influences multiple physiological processes, including bone metabolism, vascular homeostasis, and mesenchymal stem cell differentiation [17,18] (Figure 1). Dysregulation of glucocorticoid signaling has been implicated in the pathogenesis of ONFH, particularly in steroid-associated cases, where enhanced adipogenesis, suppressed osteogenesis, and vascular compromise contribute to disease progression [19,20]. Genetic polymorphisms in *NR3C1* have been associated with inter-individual variability in glucocorticoid sensitivity and have been linked to a range of diseases, including metabolic disorders, inflammatory conditions, and psychiatric disorders [21,22]. However, despite its strong biological relevance, the association between *NR3C1* genetic variants and susceptibility to ONFH has not been systematically investigated.

Therefore, the present study aimed to evaluate the association between *NR3C1* gene polymorphisms and the risk of non-traumatic ONFH using a case–control design. Given the heterogeneity of ONFH and the absence of detailed etiological exposure data, this study focuses on genetic susceptibility at the population level. By examining multiple SNPs within a biologically plausible candidate gene, this study seeks to provide exploratory insights into the genetic architecture of ONFH and to identify potential candidate variants for future risk stratification studies.

## 2. Materials and Methods

### 2.1. Study Design and Data Source

This study employed a hospital-based case–control design using genotype and clinical data obtained from the Precision Medicine Project at China Medical University Hospital (CMUH), Taiwan. All participants were aged ≥20 years and had provided written informed consent for genetic research. Peripheral venous blood samples were collected for genotyping, and clinical diagnoses were recorded using the International Classification of Diseases, Ninth Revision (ICD-9) and Tenth Revision (ICD-10).

### 2.2. Study Population

Patients with ONFH were identified using ICD-9 code 733.42 and/or ICD-10 code M87.05 between January 2003 and January 2023. To focus on non-traumatic ONFH, individuals with trauma-related diagnoses, including hip fracture or dislocation, were excluded. Control subjects were defined as individuals without diagnostic codes for ONFH throughout the study period. Because routine imaging screening was not performed in control subjects, the possibility that a small number of asymptomatic or undiagnosed ONFH cases were included in the control group cannot be excluded. Such potential outcome misclassification would likely bias the results toward the null hypothesis and reduce the ability to detect true associations, rather than exaggerating the observed associations.

Each ONFH case was matched with four control subjects based on sex and age group (≤40 years or >40 years), resulting in a 1:4 case–control ratio. Due to the retrospective nature of the dataset, detailed information on specific risk factors—such as corticosteroid exposure, alcohol consumption, smoking status, and metabolic comorbidities—was not available. Because these variables are well-established contributors to ONFH pathogenesis, residual confounding and exposure-related bias cannot be excluded.

### 2.3. Genotyping and SNP Selection

Genotyping was performed using the Taiwan Biobank version 2 (TWBv2) custom array (Thermo Fisher Scientific, Santa Clara, CA, USA), which contains approximately 690,000 markers aligned to the GRCh38 reference genome, including coding and protein-altering variants [23,24].

A total of 49 SNPs located within or near the *NR3C1* gene were selected based on their availability on the array, minor allele frequency (MAF) > 5% in the study population, and potential biological relevance to glucocorticoid signaling pathways based on prior literature. Genomic coordinates and variant annotations were obtained from the National Center for Biotechnology Information (NCBI) database.

### 2.4. Quality Control of Genotyping Data

Quality control (QC) procedures were conducted using the Axiom™ Analysis Suite (Thermo Fisher Scientific, Santa Clara, CA, USA) following the manufacturer’s recommendations. The QC process included sample-level, marker-level, and batch-level assessments to ensure genotyping reliability and data integrity.

At the SNP level, markers were evaluated based on call rate, minor allele frequency (MAF), and Hardy–Weinberg equilibrium (HWE) in the control group. SNPs with call rates < 95% or MAF < 5% were excluded from further analysis. HWE was evaluated as an additional quality-control measure to identify potential deviations in genotype distribution. SNPs showing marked deviation from HWE were reviewed and interpreted cautiously in subsequent analyses rather than automatically excluded.

Additional QC filtering for the present candidate-gene analysis was applied on a per-SNP basis. SNPs with insufficient genotype-call quality at a given locus were excluded only from the corresponding SNP-level analysis rather than through a single overall sample exclusion step. Because genotyping success and genotype-call quality may vary across loci, the effective number of analyzed individuals differed slightly among SNPs, whereas some loci retained the full cohort size. Accordingly, only participants with valid genotype calls meeting the locus-specific inclusion criteria were included in the corresponding SNP-level analyses. Therefore, the effective sample size for each SNP analysis is reflected in the genotype counts reported in the corresponding association analyses and Supplementary Table S1. No substantial batch- or plate-related artifacts were identified during QC procedures.

### 2.5. Statistical Analysis

Statistical analyses were performed by the investigators in consultation with an independent biostatistical analyst using PLINK version 1.9 (Harvard University, Cambridge, MA, USA) and R software version 4.4.2 (R Foundation for Statistical Computing, Vienna, Austria). Continuous variables were compared using Student’s *t*-test, while categorical variables were analyzed using the chi-square test.

Associations between SNPs and susceptibility to ONFH were evaluated using logistic regression models, with odds ratios (ORs) and 95% confidence intervals (CIs) estimated after adjustment for age and sex. Multiple inheritance models, including dominant, recessive, additive, and co-dominant models, were examined.

Given the number of SNPs and inheritance models evaluated, the possibility of false-positive findings due to multiple comparisons should be acknowledged. However, because this study was designed as an exploratory, hypothesis-generating candidate-gene analysis focusing on biologically plausible variants within *NR3C1*, no formal correction for multiple testing was applied. Therefore, the reported associations should be interpreted as nominal findings requiring independent replication and functional validation. Furthermore, statistical significance observed in the present study should not be interpreted as evidence of direct biological causality.

### 2.6. Ethical Approval

This study was approved by the Institutional Review Board of China Medical University Hospital (CMUH) (approval number: CMUH112-REC3-014). All participants provided written informed consent prior to enrollment.

### 2.7. Data Availability

The data used in this study were obtained from the CMUH Precision Medicine Project and are not publicly available due to institutional and ethical restrictions. Access to the data may be granted upon reasonable request and with permission from the relevant authorities.

### 2.8. Use of Generative Artificial Intelligence

Generative artificial intelligence tools (ChatGPT, OpenAI GPT-4) were used solely for language refinement and grammatical editing. The AI tools were not involved in study design, data analysis, interpretation of results, or generation of scientific conclusions.

## 3. Results

### 3.1. Baseline Characteristics of the Study Population

A total of 609 patients with ONFH and 2436 control subjects were included in this study. Cases and controls were matched at a 1:4 ratio based on sex and age group.

The ONFH group comprised 332 males (54.5%) and 277 females (45.5%), while the control group included 1330 males (54.6%) and 1106 females (45.4%). No significant difference in sex distribution was observed between the two groups (*p* > 0.999).

Regarding age distribution, 112 patients (18.4%) in the ONFH group were ≤40 years old and 497 (81.6%) were >40 years old, which was identical to the control group distribution. The mean age was 55.7 ± 15.6 years in both groups (*p* = 0.988), indicating successful matching and comparability between cases and controls (Table 1).

### 3.2. Genotyping Quality Control and SNP Characteristics

A total of 609 patients with ONFH and 2436 control subjects were included in the study cohort. After genotyping QC, all 49 selected *NR3C1* SNPs were retained for analysis. However, because genotype call quality varied across loci, some participants had missing or QC-failed genotype calls for specific SNPs. Consequently, the effective number of cases and controls included in each SNP-level association analysis varied slightly across loci. The per-SNP numbers of successfully analyzed participants are reflected in the genotype counts presented in Table 2 and Table 3.

### 3.3. Allele Frequency Distribution of NR3C1 Polymorphisms

Among the 49 SNPs analyzed, two SNPs—rs28593206 and rs315199—demonstrated nominally significant differences in allele frequency distribution between ONFH patients and control subjects. The minor allele frequencies (MAFs) of rs28593206 and rs315199 were higher in the ONFH group compared to controls and demonstrated nominally significant differences in allele frequency distribution (*p* = 0.024 and *p* = 0.009, respectively).

The remaining SNPs did not demonstrate nominally significant differences in allele frequency distribution between groups (Table 2).

### 3.4. Association Between NR3C1 Polymorphisms and ONFH Risk

Logistic regression analyses were performed to evaluate the association between *NR3C1* polymorphisms and susceptibility to ONFH under multiple genetic inheritance models. Exploratory logistic regression analyses identified nominal associations between several *NR3C1* polymorphisms and ONFH susceptibility under selected inheritance models. Among the analyzed variants, ten SNPs demonstrated nominal significance in at least one inheritance model and were therefore summarized in Table 3. Most identified variants were associated with increased odds of ONFH, with adjusted odds ratios ranging from 1.25 to 1.62. In contrast, rs7709864 was associated with reduced odds of ONFH (adjusted OR = 0.82, 95% CI: 0.70–0.97, *p* = 0.018).

However, several effect sizes were modest and accompanied by relatively wide confidence intervals. In addition, because multiple SNPs and inheritance models were evaluated without formal correction for multiple testing, the observed associations should be interpreted cautiously and considered exploratory pending independent replication. A summary of SNPs demonstrating nominal significance is presented in Table 3, while the complete genotype-based association analyses are provided in Supplementary Table S1.

### 3.5. Age-Stratified Analysis

Age-stratified analyses were subsequently performed to further evaluate potential age-related genetic effects. Nominally significant associations were more frequently observed in individuals older than 40 years of age. In this subgroup, six SNPs (rs7709864, rs890710, rs17413459, rs28593206, rs315199, and rs12658672) demonstrated nominally significant associations with ONFH susceptibility under various inheritance models (Table 4).

In contrast, no nominally significant associations were identified among participants aged 40 years or younger. However, the younger subgroup was considerably smaller, and the absence of significant findings may partly reflect limited statistical power rather than the absence of true genetic associations. Therefore, the observed age-related differences should be interpreted cautiously. Overall, these findings suggest that the influence of *NR3C1* polymorphisms on ONFH susceptibility may vary across age groups and warrants further investigation in larger and independent cohorts.

## 4. Discussion

This study suggests that polymorphisms in the *NR3C1* gene may be associated with susceptibility to non-traumatic osteonecrosis of the femoral head (ONFH). Several variants showed nominal associations under different genetic models, and these signals appeared more evident among individuals older than 40 years. However, these findings should be interpreted as exploratory and hypothesis-generating rather than definitive evidence of genetic susceptibility, because multiple SNPs and inheritance models were tested without formal correction for multiple comparisons. Therefore, although the results support *NR3C1* as a biologically plausible candidate gene in ONFH, they require cautious interpretation and independent validation.

The pathogenesis of ONFH is complex and multifactorial, involving vascular impairment, altered lipid metabolism, inflammation, adipogenic differentiation, and dysregulated bone remodeling [25,26,27,28]. Previous pathological and experimental studies have demonstrated that ischemia, osteocyte apoptosis, marrow fat accumulation, and impaired microcirculation are central mechanisms contributing to femoral head necrosis [29,30,31]. Glucocorticoid exposure is one of the major contributors to non-traumatic ONFH and may promote disease development by enhancing adipogenesis, suppressing osteogenesis, and impairing vascular homeostasis [2,20]. The *NR3C1* gene encodes the glucocorticoid receptor (GR), which mediates cellular responses to glucocorticoids and regulates downstream transcriptional programs relevant to bone and vascular biology [32]. Previous studies have suggested that *NR3C1* polymorphisms may influence glucocorticoid sensitivity and transcriptional activity, contributing to inter-individual differences in metabolic, inflammatory, and skeletal phenotypes. In this context, the observed nominal associations between *NR3C1* variants and ONFH risk are biologically plausible, although causality cannot be inferred from the present data.

Although genome-wide association studies (GWAS) provide an unbiased strategy for variant discovery, hypothesis-driven candidate-gene approaches remain valuable when investigating biologically relevant pathways with established mechanistic links to disease pathogenesis. Given the known role of glucocorticoid signaling in bone metabolism, vascular homeostasis, and steroid-associated ONFH, exploration of *NR3C1* polymorphisms represents a biologically plausible approach for identifying potential susceptibility variants.

In the present study, rs28593206 and rs315199 were associated with increased odds of ONFH, whereas rs7709864 demonstrated a potential protective association. Nevertheless, the biological interpretation of these variants remains limited. Current evidence does not conclusively establish rs28593206, rs315199, or rs7709864 as functional variants directly affecting glucocorticoid receptor expression, ligand binding, or downstream signaling activity. In addition, evidence linking these SNPs to altered *NR3C1* expression in bone, vascular tissue, mesenchymal stem cells, or femoral head tissue remains insufficient. Because these variants are primarily located in non-coding regions and are not known to directly alter protein structure, their potential effects may involve regulatory mechanisms, linkage disequilibrium with nearby functional loci, or context-dependent gene–environment interactions. Further studies incorporating expression quantitative trait locus (eQTL) analyses, chromatin annotation, reporter assays, and glucocorticoid-response functional experiments are required to clarify their biological relevance in ONFH pathogenesis. Although two SNPs demonstrated marked deviation from HWE in the control group, neither variant was among the primary nominally associated SNPs highlighted in the main analyses. Therefore, the main exploratory findings were not driven by variants with marked HWE deviation.

The age-stratified findings are also noteworthy but should be interpreted cautiously. Significant associations were mainly observed among individuals older than 40 years, whereas no significant associations were detected in younger participants. This pattern may reflect age-related changes in glucocorticoid receptor expression, receptor sensitivity, vascular function, bone remodeling capacity, or cumulative environmental exposures. However, the younger subgroup was considerably smaller, and the absence of significant associations in younger individuals may partly reflect reduced statistical power rather than true absence of genetic effect. Therefore, the apparent age-dependent association should be regarded as an exploratory observation requiring confirmation in larger cohorts with sufficient sample size across age strata.

From a clinical perspective, ONFH is important because it often affects young and middle-aged adults and may progress to femoral head collapse, loss of hip function, and early total hip arthroplasty. Earlier identification of individuals at increased risk could potentially support closer surveillance and joint-preserving interventions before end-stage joint destruction. However, the present findings are not yet clinically actionable. They should not be used for genetic screening or individual risk prediction until replicated in independent cohorts and supported by functional evidence. The long-term consequences of arthroplasty are particularly relevant in younger ONFH patients, who may face prolonged implant survival demands and possible revision surgery. Historically, some patients underwent metal-on-metal total hip arthroplasty, which has been associated with metal ion release, particularly cobalt and chromium, adverse local reactions to metal debris, and potential systemic concerns; therefore, long-term monitoring of such patients has been recommended. These considerations further emphasize the importance of preventing disease progression and preserving the native joint whenever feasible [4].

Several limitations of this study should be acknowledged. First, this was a hospital-based case–control study, and control subjects were defined by the absence of ONFH diagnostic codes. Although trauma-related diagnoses were excluded, detailed etiological information regarding corticosteroid exposure, corticosteroid dosage, alcohol consumption, smoking status, metabolic disorders, autoimmune diseases, and other established ONFH risk factors was unavailable. Therefore, stratified analyses according to specific ONFH etiologies, such as steroid-associated, alcohol-associated, idiopathic, or other subtypes, could not be performed. Because these environmental and clinical factors are central to ONFH pathogenesis and may potentially correlate with genotype distribution, residual confounding, unmeasured environmental influences, and spurious associations cannot be excluded. This critical shortcoming limits causal inference, and the present results alone cannot justify changes in clinical practice. Second, non-traumatic ONFH represents a heterogeneous disease entity, and the absence of etiological stratification prevented determination of whether the observed associations were specific to steroid-associated, alcohol-associated, idiopathic, or other ONFH subtypes. Third, given the number of SNPs and inheritance models evaluated, the risk of type I error due to multiple comparisons must be acknowledged. Because no formal correction for multiple testing was applied, some nominally significant findings may represent false-positive associations rather than true genetic effects. However, this study was designed as an exploratory, hypothesis-generating candidate-gene analysis focusing on biologically plausible *NR3C1* variants, and overly stringent correction methods may increase the risk of type II error and obscure potentially relevant signals for future investigation. Therefore, all reported associations should be interpreted cautiously as preliminary, nominal findings requiring validation in independent cohorts. Fourth, replication in an independent cohort was not performed, which further limits the robustness and generalizability of the findings. Finally, no direct functional or histopathological validation was conducted, and the biological significance of the identified variants therefore remains uncertain. Despite these limitations, this study provides exploratory evidence supporting *NR3C1* as a biologically plausible candidate gene in ONFH susceptibility. The findings may complement genome-wide approaches by focusing on pathways directly relevant to glucocorticoid signaling, osteogenesis, adipogenesis, and vascular regulation. Future studies should validate these associations in larger, multi-center cohorts with detailed clinical exposure data and should incorporate etiological subgroup analyses, gene–environment interaction testing, and functional investigations to clarify the biological relevance of the identified variants. Importantly, before any clinical application of *NR3C1* genotyping for risk stratification or personalized prevention can be considered, multivariate risk prediction models integrating genetic variants with environmental and clinical factors—such as corticosteroid exposure, alcohol consumption, smoking status, metabolic comorbidities, and other established ONFH risk factors—will be required.

## 5. Conclusions

In conclusion, this exploratory study suggests that genetic polymorphisms in the *NR3C1* gene may be associated with susceptibility to non-traumatic osteonecrosis of the femoral head (ONFH), with findings indicating a potential age-dependent effect, particularly among individuals over 40 years of age. These observations further support the biological relevance of glucocorticoid signaling in ONFH pathogenesis and identify *NR3C1* as a biologically plausible candidate gene that may contribute to disease susceptibility.

However, given the exploratory nature of this study, the absence of detailed clinical exposure data, and the lack of correction for multiple testing, the reported associations should be interpreted with caution. The findings require independent replication in larger, well-characterized cohorts with comprehensive clinical and etiological information, as well as functional validation studies, before any potential clinical implications can be considered.

Despite these limitations, this study provides preliminary exploratory insights into the potential genetic architecture of ONFH and highlights the importance of integrating genetic factors into the understanding of disease susceptibility. Future studies incorporating gene–environment interactions, replication analyses, and functional investigations will be necessary to clarify the role of *NR3C1* polymorphisms in ONFH pathogenesis.

## Figures and Tables

**Table 1:** Demographic characteristics of participants with or without ONFH.

	ONFH	Control	*p*-Value
Sex			>0.999
Male	332	1330	
Female	277	1106	
Age group			>0.999
≤40 years	112	448	
>40 years	497	1988	
Age (years)			
Mean ± SD	55.7 ± 15.6	55.7 ± 15.6	0.988

**Table 2:** Genotyping of *NR3C1* polymorphisms in patients with ONFH and control subjects.

SNP	Chromosome: Position	Allele	MAF ^1^		HWE ^2^ *p*-Value	OR (95%CI)	*p*-Value
			Case	Control			
rs6191	5:143278591	A/C	0.262712	0.267241	0.402261	0.98 (0.62, 1.55)	0.921
rs6196	5:143281925	G/A	0.08046	0.068817	0.709566	1.18 (0.94, 1.50)	0.158
rs258751	5:143282715	A/G	0.079639	0.068173	0.678155	1.18 (0.93, 1.50)	0.162
rs6194	5:143298796	A/G	0.079639	0.067256	0.735	1.20 (0.95, 1.52)	0.129
rs79590198	5:143307371	A/G	0.136513	0.142475	0.837956	0.95 (0.79, 1.14)	0.593
rs852977	5:143307929	G/A	0.107908	0.101527	0.087811	1.07 (0.87, 1.31)	0.513
rs2963155	5:143376439	G/A	0.179803	0.179745	0.177932	1.00 (0.85, 1.18)	0.996
rs9324921	5:143388175	A/C	0.237232	0.225773	0.451398	1.07 (0.92, 1.24)	0.395
rs11749561	5:143412112	C/T	0.115702	0.113009	0.915717	1.03 (0.84, 1.25)	0.792
rs7709864	5:143443564	C/T	0.218647	0.238496	0.437058	0.89 (0.77, 1.04)	0.145
rs34125834	5:143447752	GA/G	0.304778	0.298144	0.064922	1.03 (0.90, 1.18)	0.652
rs11167813	5:143469539	G/T	0.434319	0.422602	0.656191	1.05 (0.92, 1.19)	0.460
rs17400549	5:143474256	C/T	0.079639	0.090816	0.192486	0.87 (0.69, 1.09)	0.220
rs17100326	5:143483391	T/C	0.067545	0.068817	0.312277	0.98 (0.76, 1.26)	0.875
rs1374001	5:143494412	G/T	0.09688	0.109285	0.055595	0.87 (0.71, 1.08)	0.210
rs12153243	5:143520236	T/A	0.192771	0.204222	1.34 × 10^−9^	0.93 (0.79, 1.09)	0.385
rs11167820	5:143536876	A/G	0.370066	0.374435	0.477741	0.98 (0.86, 1.12)	0.778
rs6893954	5:143537442	A/G	0.413793	0.405339	0.911734	1.04 (0.91, 1.18)	0.591
rs4912927	5:143571529	G/A	0.485149	0.484961	0.906566	1.00 (0.88, 1.14)	0.991
rs17100411	5:143586103	C/A	0.236453	0.236712	0.298097	1.00 (0.86, 1.16)	0.985
rs153516	5:143599618	G/A	0.49341	0.497736	0.934903	0.98 (0.87, 1.11)	0.788
rs1582888	5:143604152	G/A	0.111658	0.112777	0.584303	0.99 (0.81, 1.21)	0.912
rs17404863	5:143624083	C/T	0.206755	0.219136	0.969948	0.93 (0.80, 1.08)	0.349
rs247623	5:143630147	G/T	0.085667	0.096296	0.604605	0.88 (0.70, 1.10)	0.257
rs12659355	5:143630724	C/T	0.3	0.282094	0.32686	1.09 (0.95, 1.25)	0.218
rs11738413	5:143637655	C/T	0.336634	0.34075	5.13 × 10^−16^	0.98 (0.86, 1.12)	0.787
rs56318527	5:143646240	C/T	0.362524	0.355649	0.253207	1.03 (0.90, 1.18)	0.675
rs10063590	5:143648776	G/T	0.362438	0.377218	0.345076	0.94 (0.82, 1.07)	0.341
rs1213706	5:143651416	G/A	0.179868	0.186266	0.643873	0.96 (0.81, 1.13)	0.608
rs2910256	5:143654948	A/G	0.506601	0.486587	0.157098	1.08 (0.96, 1.23)	0.213
rs1427864	5:143657295	C/A	0.331405	0.335346	0.039188	0.98 (0.86, 1.12)	0.795
rs890710	5:143666973	T/C	0.303777	0.278166	0.389696	1.13 (0.99, 1.30)	0.076
rs10061924	5:143683516	G/A	0.381773	0.38107	0.56982	1.00 (0.88, 1.14)	0.964
rs12717905	5:143694820	A/G	0.476936	0.4905	0.751696	0.95 (0.84, 1.07)	0.398
rs17413459	5:143699643	A/G	0.123153	0.105955	0.853518	1.18 (0.98, 1.44)	0.085
rs6862531	5:143706388	A/G	0.123558	0.106247	0.692384	1.19 (0.98, 1.44)	0.084
rs74567155	5:143734935	C/CCTT	0.314356	0.3264	0.921749	0.95 (0.83, 1.08)	0.423
rs116972667	5:143757295	A/G	0.055008	0.048991	0.594591	1.13 (0.86, 1.49)	0.390
rs1387791	5:143772976	C/T	0.28089	0.269975	0.60079	1.06 (0.92, 1.22)	0.445
rs10515524	5:143793129	A/G	0.454545	0.450928	0.035307	1.01 (0.89, 1.15)	0.821
rs3805462	5:143813893	G/T	0.376847	0.383855	0.243327	0.97 (0.85, 1.10)	0.653
rs12519988	5:143832024	T/C	0.161157	0.176349	0.387085	0.90 (0.76, 1.06)	0.212
rs13165784	5:143844513	T/C	0.133224	0.121249	0.896794	1.11 (0.92, 1.34)	0.257
rs12518693	5:143860522	G/T	0.183388	0.203003	0.23098	0.88 (0.75, 1.04)	0.126
rs7717240	5:143872027	T/C	0.145799	0.147483	0.246642	0.99 (0.83, 1.18)	0.882
rs28593206	5:143872642	A/G	0.463636	0.427716	0.465969	1.16 (1.02, 1.31)	0.024 *
rs315180	5:143910684	A/G	0.126645	0.127673	0.507388	0.99 (0.82, 1.20)	0.923
rs315199	5:143929003	A/G	0.416804	0.376287	0.995649	1.18 (1.04, 1.35)	0.009 **
rs12658672	5:143952976	A/G	0.230198	0.204959	0.762071	1.16 (1.00, 1.35)	0.054

^1^ Minor allele frequency; ^2^ Hardy–Weinberg equilibrium; * *p* < 0.05, ** *p* < 0.01, The number of analyzed individuals may vary slightly across SNPs because genotype calls failing locus-specific QC were excluded from the corresponding SNP-level analysis.

**Table 3:** Summary of nominally significant associations between *NR3C1* polymorphisms and ONFH susceptibility.

SNP	Model	Genotype	ONFH	Control	cOR ^1^ (95%CI)	*p*-Value	aOR ^2^ (95%CI)	*p*-Value
rs6196	Co-dominant	AA	518	2109	1.00 (ref.)	—	1.00 (ref.)	—
		GG	84	315	2.85 (1.08, 7.52)	0.034 *	2.89 (1.10, 7.64)	0.032 *
		AG	7	10	1.09 (0.84, 1.41)	0.534	1.07 (0.82, 1.39)	0.625
	Dominant	AA	518	2109	1.00 (ref.)	—	1.00 (ref.)	—
		GG + AG	91	325	1.14 (0.89, 1.47)	0.307	1.12 (0.87, 1.45)	0.369
	Recessive	GG	84	315	1.00 (ref.)	—	1.00 (ref.)	—
		AG + AA	525	2119	2.82 (1.07, 7.44)	0.036 *	2.87 (1.09, 7.56)	0.033 *
	Additive	—	—	—	1.18 (0.94, 1.49)	0.160	1.17 (0.92, 1.48)	0.194
rs258751	Co-dominant	GG	519	2112	1.00 (ref.)	—	1.00 (ref.)	—
		AA	83	314	3.17 (1.17, 8.54)	0.023 *	3.21 (1.19, 8.66)	0.021 *
		GA	7	9	1.08 (0.83, 1.40)	0.583	1.06 (0.81, 1.37)	0.678
	Dominant	GG	519	2112	1.00 (ref.)	—	1.00 (ref.)	—
		AA + GA	90	323	1.13 (0.88, 1.46)	0.330	1.12 (0.87, 1.44)	0.395
	Recessive	AA	83	314	1.00 (ref.)	—	1.00 (ref.)	—
		GA + GG	526	2121	3.13 (1.16, 8.45)	0.024 *	3.19 (1.18, 8.60)	0.022 *
	Additive	—	—	—	1.18 (0.93, 1.50)	0.163	1.17 (0.92, 1.48)	0.200
rs6194	Co-dominant	GG	519	2112	1.00 (ref.)	—	1.00 (ref.)	—
		AA	83	311	3.56 (1.29, 9.86)	0.015 *	3.61 (1.30, 10.01)	0.013 *
		GA	7	8	1.09 (0.84, 1.41)	0.535	1.07 (0.82, 1.39)	0.627
	Dominant	GG	519	2112	1.00 (ref.)	—	1.00 (ref.)	—
		AA + GA	90	319	1.15 (0.89, 1.48)	0.284	1.13 (0.88, 1.46)	0.345
	Recessive	AA	83	311	1.00 (ref.)	—	1.00 (ref.)	—
		GA + GG	526	2120	3.52 (1.27, 9.75)	0.015 *	3.58 (1.29, 9.92)	0.014 *
	Additive	—	—	—	1.20 (0.95, 1.52)	0.130	1.19 (0.93, 1.50)	0.161
rs7709864	Co-dominant	TT	377	1414	1.00 (ref.)	—	1.00 (ref.)	—
		CC	193	879	0.96 (0.65, 1.40)	0.825	0.94 (0.64, 1.38)	0.742
		TC	36	141	0.82 (0.68, 1.00)	0.048 *	0.81 (0.67, 0.98)	0.033 *
	Dominant	TT	377	1414	1.00 (ref.)	—	1.00 (ref.)	—
		CC + TC	229	1020	0.84 (0.70, 1.01)	0.065	0.83 (0.69, 0.99)	0.043 *
	Recessive	CC	193	879	1.00 (ref.)	—	1.00 (ref.)	—
		TC + TT	413	1555	1.03 (0.70, 1.50)	0.890	1.01 (0.69, 1.48)	0.958
	Additive	—	—	—	0.90 (0.77, 1.04)	0.148	0.88 (0.76, 1.03)	0.104
rs890710	Co-dominant	CC	284	1270	1.00 (ref.)	—	1.00 (ref.)	—
		TT	280	971	1.05 (0.75, 1.49)	0.767	1.07 (0.75, 1.51)	0.715
		CT	45	191	1.29 (1.07, 1.55)	0.007 **	1.28 (1.06, 1.54)	0.010 *
	Dominant	CC	284	1270	1.00 (ref.)	—	1.00 (ref.)	—
		TT + CT	325	1162	1.25 (1.05, 1.49)	0.013 *	1.24 (1.04, 1.48)	0.018 *
	Recessive	TT	280	971	1.00 (ref.)	—	1.00 (ref.)	—
		CT + CC	329	1461	0.94 (0.67, 1.31)	0.699	0.95 (0.68, 1.33)	0.779
	Additive	—	—	—	1.13 (0.99, 1.30)	0.074	1.13 (0.99, 1.30)	0.080
rs17413459	Co-dominant	GG	463	1947	1.00 (ref.)	—	1.00 (ref.)	—
		AA	142	460	0.60 (0.21, 1.72)	0.343	0.61 (0.21, 1.75)	0.356
		GA	4	28	1.30 (1.05, 1.61)	0.017 *	1.28 (1.03, 1.58)	0.027 *
	Dominant	GG	463	1947	1.00 (ref.)	—	1.00 (ref.)	—
		AA + GA	146	488	1.26 (1.02, 1.55)	0.033 *	1.24 (1.00, 1.53)	0.049 *
	Recessive	AA	142	460	1.00 (ref.)	—	1.00 (ref.)	—
		GA + GG	467	1975	0.57 (0.20, 1.63)	0.292	0.58 (0.20, 1.66)	0.307
	Additive	—	—	—	1.19 (0.98, 1.44)	0.084	1.17 (0.96, 1.43)	0.112
rs6862531	Co-dominant	GG	461	1944	1.00 (ref.)	—	1.00 (ref.)	—
		AA	142	461	0.60 (0.21, 1.73)	0.345	0.61 (0.21, 1.75)	0.359
		GA	4	28	1.30 (1.05, 1.61)	0.016 *	1.28 (1.03, 1.58)	0.026 *
	Dominant	GG	461	1944	1.00 (ref.)	—	1.00 (ref.)	—
		AA + GA	146	489	1.26 (1.02, 1.55)	0.032 *	1.24 (1.00, 1.53)	0.048 *
	Recessive	AA	142	461	1.00 (ref.)	—	1.00 (ref.)	—
		GA + GG	465	1972	0.57 (0.20, 1.63)	0.294	0.58 (0.20, 1.66)	0.309
	Additive	—	—	—	1.19 (0.98, 1.45)	0.082	1.17 (0.96, 1.43)	0.110
rs28593206	Co-dominant	GG	168	789	1.00 (ref.)	—	1.00 (ref.)	—
		AA	313	1193	1.33 (1.02, 1.72)	0.033 *	1.34 (1.03, 1.74)	0.027 *
		GA	124	439	1.23 (1.00, 1.52)	0.049 *	1.24 (1.00, 1.52)	0.047 *
	Dominant	GG	168	789	1.00 (ref.)	—	1.00 (ref.)	—
		AA + GA	437	1632	1.26 (1.03, 1.53)	0.023 *	1.26 (1.04, 1.54)	0.020 *
	Recessive	AA	313	1193	1.00 (ref.)	—	1.00 (ref.)	—
		GA + GG	292	1228	1.16 (0.93, 1.45)	0.182	1.18 (0.94, 1.47)	0.156
	Additive	—	—	—	1.16 (1.02, 1.32)	0.023 *	1.17 (1.03, 1.32)	0.019 *
rs315199	Co-dominant	GG	206	945	1.00 (ref.)	—	1.00 (ref.)	—
		AA	296	1140	1.40 (1.07, 1.83)	0.013 *	1.42 (1.09, 1.85)	0.010 *
		GA	105	344	1.19 (0.98, 1.45)	0.083	1.19 (0.98, 1.45)	0.087
	Dominant	GG	206	945	1.00 (ref.)	—	1.00 (ref.)	—
		AA + GA	401	1484	1.24 (1.03, 1.49)	0.024 *	1.24 (1.03, 1.50)	0.023 *
	Recessive	AA	296	1140	1.00 (ref.)	—	1.00 (ref.)	—
		GA + GG	311	1289	1.27 (1.00, 1.61)	0.052	1.29 (1.01, 1.64)	0.039 *
	Additive	—	—	—	1.18 (1.04, 1.35)	0.010 *	1.19 (1.05, 1.35)	0.008 **
rs12658672	Co-dominant	GG	366	1531	1.00 (ref.)	—	1.00 (ref.)	—
		AA	201	786	1.58 (1.08, 2.33)	0.019 *	1.62 (1.10, 2.38)	0.015 *
		GA	39	103	1.07 (0.88, 1.30)	0.492	1.08 (0.89, 1.31)	0.442
	Dominant	GG	366	1531	1.00 (ref.)	—	1.00 (ref.)	—
		AA + GA	240	889	1.13 (0.94, 1.36)	0.191	1.14 (0.95, 1.37)	0.159
	Recessive	AA	201	786	1.00 (ref.)	—	1.00 (ref.)	—
		GA + GG	405	1634	1.55 (1.06, 2.26)	0.024 *	1.57 (1.08, 2.30)	0.019 *
	Additive	—	—	—	1.16 (1.00, 1.34)	0.056	1.17 (1.01, 1.36)	0.042 *

^1^ Crude odds ratio. ^2^ Adjusted odds ratio (adjusted for age and sex). * *p* < 0.05, ** *p* < 0.01, and —: not applicable. No formal correction for multiple testing was applied because this study was designed as an exploratory, hypothesis-generating candidate-gene analysis. Therefore, the reported associations should be interpreted as nominal findings requiring independent validation. Genotype counts represent the number of participants with valid genotype calls for each SNP and inheritance model. Because locus-specific quality-control filtering was applied, the effective sample size varied slightly across SNPs.

**Table 4:** Age-stratified analysis of *NR3C1* polymorphisms and the risk of osteonecrosis of the femoral head (ONFH) under different genetic models.

			>40 Years				≤40 Years			
SNP	Model	Genotype	Case	Control	aOR ^1^ (95%CI)	*p*-Value	Case	Control	aOR ^2^ (95%CI)	*p*-Value
rs6196	Allele	A	916	3701	1.00 (ref.)	—	204	832	1.00 (ref.)	—
		G	78	271	1.16 (0.89, 1.51)	0.259	20	64	1.27 (0.75, 2.15)	0.364
	Co-dominant	AA	424	1724	1.00 (ref.)	—	94	385	1.00 (ref.)	—
		GG	68	253	2.26 (0.75, 6.78)	0.146	16	62	8.19 (0.73, 91.30)	0.087
		AG	5	9	1.09 (0.82, 1.46)	0.546	2	1	1.06 (0.58, 1.91)	0.855
	Dominant	AA	424	1724	1.00 (ref.)	—	94	385	1.00 (ref.)	—
		GG + AG	73	262	1.13 (0.86, 1.50)	0.383	18	63	1.17 (0.66, 2.07)	0.589
	Recessive	GG	68	253	1.00 (ref.)	—	16	62	1.00 (ref.)	—
		AG + AA	429	1733	2.23 (0.74, 6.69)	0.152	96	386	8.13 (0.73, 90.45)	0.088
	Additive	—	—	—	1.16 (0.89, 1.51)	0.262	—	—	1.28 (0.75, 2.16)	0.364
rs258751	Allele	G	917	3706	1.00 (ref.)	—	204	832	1.00 (ref.)	—
		A	77	268	1.16 (0.89, 1.51)	0.266	20	64	1.27 (0.75, 2.15)	0.364
	Co-dominant	GG	425	1727	1.00 (ref.)	—	94	385	1.00 (ref.)	—
		AA	67	252	2.54 (0.83, 7.80)	0.104	16	62	8.19 (0.73, 91.30)	0.087
		GA	5	8	1.08 (0.81, 1.44)	0.601	2	1	1.06 (0.58, 1.91)	0.855
	Dominant	GG	425	1727	1.00 (ref.)	—	94	385	1.00 (ref.)	—
		AA + GA	72	260	1.13 (0.85, 1.49)	0.412	18	63	1.17 (0.66, 2.07)	0.589
	Recessive	AA	67	252	1.00 (ref.)	—	16	62	1.00 (ref.)	—
		GA + GG	430	1735	2.51 (0.82, 7.72)	0.107	96	386	8.13 (0.73, 90.45)	0.088
	Additive	—	—	—	1.16 (0.89, 1.51)	0.268	—	—	1.28 (0.75, 2.16)	0.364
rs6194	Allele	G	917	3703	1.00 (ref.)	—	204	832	1.00 (ref.)	—
		A	77	265	1.17 (0.90, 1.53)	0.235	20	62	1.32 (0.78, 2.23)	0.306
	Co-dominant	GG	425	1726	1.00 (ref.)	—	94	386	1.00 (ref.)	—
		AA	67	251	2.90 (0.92, 9.18)	0.070	16	60	8.21 (0.74, 91.54)	0.087
		GA	5	7	1.08 (0.81, 1.45)	0.585	2	1	1.10 (0.60, 1.99)	0.765
	Dominant	GG	425	1726	1.00 (ref.)	—	94	386	1.00 (ref.)	—
		AA + GA	72	258	1.13 (0.86, 1.50)	0.382	18	61	1.21 (0.68, 2.15)	0.510
	Recessive	AA	67	251	1.00 (ref.)	—	16	60	1.00 (ref.)	—
		GA + GG	430	1733	2.87 (0.91, 9.08)	0.073	96	387	8.11 (0.73, 90.24)	0.089
	Additive	—	—	—	1.17 (0.90, 1.53)	0.235	—	—	1.32 (0.78, 2.23)	0.308
rs7709864	Allele	T	775	3038	1.00 (ref.)	—	172	669	1.00 (ref.)	—
		C	215	934	0.90 (0.76, 1.07)	0.230	50	227	0.86 (0.60, 1.22)	0.385
	Co-dominant	TT	311	1162	1.00 (ref.)	—	66	252	1.00 (ref.)	—
		CC	153	714	1.05 (0.69, 1.60)	0.808	40	165	0.62 (0.24, 1.60)	0.320
		TC	31	110	0.80 (0.65, 0.99)	0.042 *	5	31	0.93 (0.60, 1.44)	0.730
	Dominant	TT	311	1162	1.00 (ref.)	—	66	252	1.00 (ref.)	—
		CC + TC	184	824	0.83 (0.68, 1.02)	0.080	45	196	0.88 (0.58, 1.33)	0.539
	Recessive	CC	153	714	1.00 (ref.)	—	40	165	1.00 (ref.)	—
		TC + TT	342	1272	1.14 (0.76, 1.72)	0.532	71	283	0.63 (0.25, 1.63)	0.344
	Additive	—	—	—	0.90 (0.77, 1.07)	0.235	—	—	0.86 (0.61, 1.21)	0.389
rs890710	Allele	C	692	2866	1.00 (ref.)	—	156	645	1.00 (ref.)	—
		T	302	1102	1.14 (0.97, 1.32)	0.102	68	251	1.12 (0.81, 1.54)	0.487
	Co-dominant	CC	236	1046	1.00 (ref.)	—	48	224	1.00 (ref.)	—
		TT	220	774	1.11 (0.77, 1.60)	0.583	60	197	0.69 (0.23, 2.07)	0.509
		CT	41	164	1.26 (1.03, 1.55)	0.028 *	4	27	1.42 (0.93, 2.17)	0.105
	Dominant	CC	236	1046	1.00 (ref.)	—	48	224	1.00 (ref.)	—
		TT + CT	261	938	1.23 (1.01, 1.50)	0.036 *	64	224	1.33 (0.88, 2.02)	0.177
	Recessive	TT	220	774	1.00 (ref.)	—	60	197	1.00 (ref.)	—
		CT + CC	277	1210	1.00 (0.70, 1.42)	0.990	52	251	0.58 (0.20, 1.69)	0.315
	Additive	—	—	—	1.13 (0.97, 1.32)	0.105	—	—	1.14 (0.81, 1.61)	0.457
rs17413459	Allele	G	871	3547	1.00 (ref.)	—	197	807	1.00 (ref.)	—
		A	123	427	1.17 (0.95, 1.45)	0.143	27	89	1.24 (0.79, 1.97)	0.352
	Co-dominant	GG	377	1585	1.00 (ref.)	—	86	362	1.00 (ref.)	—
		AA	117	377	0.50 (0.15, 1.68)	0.265	25	83	1.40 (0.14, 13.65)	0.770
		GA	3	25	1.30 (1.03, 1.65)	0.027 *	1	3	1.27 (0.77, 2.10)	0.357
	Dominant	GG	377	1585	1.00 (ref.)	—	86	362	1.00 (ref.)	—
		AA + GA	120	402	1.25 (0.99, 1.58)	0.056	26	86	1.27 (0.77, 2.09)	0.342
	Recessive	AA	117	377	1.00 (ref.)	—	25	83	1.00 (ref.)	—
		GA + GG	380	1610	0.48 (0.14, 1.58)	0.227	87	365	1.34 (0.14, 12.97)	0.803
	Additive	—	—	—	1.18 (0.95, 1.46)	0.142	—	—	1.25 (0.79, 2.00)	0.343
rs28593206	Allele	G	529	2269	1.00 (ref.)	—	120	502	1.00 (ref.)	—
		A	457	1681	1.17 (1.01, 1.34)	0.032 *	104	390	1.12 (0.83, 1.50)	0.466
	Co-dominant	GG	139	649	1.00 (ref.)	—	29	140	1.00 (ref.)	—
		AA	251	971	1.35 (1.02, 1.80)	0.037 *	62	222	1.21 (0.65, 2.25)	0.554
		GA	103	355	1.21 (0.96, 1.52)	0.109	21	84	1.35 (0.83, 2.20)	0.231
	Dominant	GG	139	649	1.00 (ref.)	—	29	140	1.00 (ref.)	—
		AA + GA	354	1326	1.25 (1.00, 1.55)	0.047 *	83	306	1.31 (0.82, 2.08)	0.256
	Recessive	AA	251	971	1.00 (ref.)	—	62	222	1.00 (ref.)	—
		GA + GG	242	1004	1.21 (0.94, 1.54)	0.136	50	224	0.99 (0.59, 1.69)	0.984
	Additive	—	—	—	1.17 (1.01, 1.34)	0.031 *	—	—	1.12 (0.83, 1.51)	0.459
rs315199	Allele	G	579	2477	1.00 (ref.)	—	129	553	1.00 (ref.)	—
		A	411	1495	1.18 (1.02, 1.35)	0.025 *	95	333	1.22 (0.91, 1.65)	0.185
	Co-dominant	GG	172	773	1.00 (ref.)	—	34	172	1.00 (ref.)	—
		AA	235	931	1.40 (1.05, 1.88)	0.023 *	61	209	1.39 (0.72, 2.66)	0.324
		GA	88	282	1.13 (0.91, 1.41)	0.258	17	62	1.48 (0.93, 2.35)	0.100
	Dominant	GG	172	773	1.00 (ref.)	—	34	172	1.00 (ref.)	—
		AA + GA	323	1213	1.20 (0.97, 1.47)	0.087	78	271	1.46 (0.93, 2.27)	0.098
	Recessive	AA	235	931	1.00 (ref.)	—	61	209	1.00 (ref.)	—
		GA + GG	260	1055	1.31 (1.00, 1.70)	0.046 *	51	234	1.10 (0.61, 1.97)	0.749
	Additive	—	—	—	1.18 (1.02, 1.35)	0.025 *	—	—	1.23 (0.91, 1.66)	0.179
rs12658672	Allele	G	766	3135	1.00 (ref.)	—	167	713	1.00 (ref.)	—
		A	222	809	1.12 (0.95, 1.33)	0.176	57	183	1.33 (0.94, 1.87)	0.101
	Co-dominant	GG	304	1244	1.00 (ref.)	—	62	287	1.00 (ref.)	—
		AA	158	647	1.62 (1.05, 2.48)	0.028 *	43	139	1.47 (0.60, 3.60)	0.396
		GA	32	81	1.00 (0.81, 1.24)	0.995	7	22	1.43 (0.92, 2.22)	0.109
	Dominant	GG	304	1244	1.00 (ref.)	—	62	287	1.00 (ref.)	—
		AA + GA	190	728	1.07 (0.87, 1.31)	0.525	50	161	1.44 (0.94, 2.19)	0.090
	Recessive	AA	158	647	1.00 (ref.)	—	43	139	1.00 (ref.)	—
		GA + GG	336	1325	1.62 (1.06, 2.47)	0.026 *	69	309	1.29 (0.54, 3.10)	0.568
	Additive	—	—	—	1.12 (0.95, 1.33)	0.179	—	—	1.32 (0.94, 1.84)	0.109

^1,2^ Adjusted odds ratio (adjusted for age and gender). * *p* < 0.05. Owing to variation in quality control pass rates among individual SNPs, the number of patients included in the final analysis varied slightly for each SNP locus.

## Data Availability

The data presented in this study are available on reasonable request from the corresponding author because the dataset contains sensitive human genetic and clinical information derived from hospital-based records. In accordance with institutional regulations, ethical approval requirements, and data protection policies, the raw data cannot be made publicly available.

## Supplementary Materials

The following supporting information can be downloaded at: https://www.mdpi.com/article/10.3390/medicina62061068/s1, Table S1. Complete exploratory genotype-based association analyses of *NR3C1* polymorphisms under multiple inheritance models.

## Abbreviations

The following abbreviations are used in this manuscript:

ONFH	Osteonecrosis of the Femoral Head
*NR3C1*	Nuclear Receptor Subfamily 3 Group C Member 1
GR	Glucocorticoid Receptor
SNP	Single-Nucleotide Polymorphism
GWAS	Genome-Wide Association Study
MAF	Minor Allele Frequency
HWE	Hardy–Weinberg Equilibrium
OR	Odds Ratio
CI	Confidence Interval
QC	Quality Control
TWBv2	Taiwan Biobank Version 2
CMUH	China Medical University Hospital
ICD-9	International Classification of Diseases-9
ICD-10	International Classification of Diseases-10
