# Supplementary material for: Glucocorticoid Receptor Gene Polymorphisms and Femoral Head Osteonecrosis
Source: Medicina (Kaunas). 2026 May 31;62(6):1068. doi: 10.3390/medicina62061068 (PMC13303425; doi:10.3390/medicina62061068)
Supplement: Supplementary file 1 [file medicina-62-01068-s001.zip › medicina-4307784-supplementary.pdf]

medicina

MDPI

Supplementary Table S1. Complete exploratory genotype-based association analyses of NR3C1 polymorphisms under multiple inheritance models.

SNP	Model	Genotype	ONFH	Control	cOR[1] (95%CI)	p-value	aOR[2] (95%CI)	p-value
rs6191	Co-dominant	CC	34	124	1.00 (ref.)	—	1.00 (ref.)	—
		AA	19	92	1.37 (0.50, 3.76)	0.544	NC (NC, NC)	NC
		CA	6	16	0.75 (0.40, 1.40)	0.372	NC (NC, NC)	NC
	Dominant	CC	34	124	1.00 (ref.)	—	1.00 (ref.)	—
		AA+CA	25	108	0.84 (0.47, 1.50)	0.565	NC (NC, NC)	NC
	Recessive	AA	19	92	1.00 (ref.)	—	1.00 (ref.)	—
		CA+CC	40	140	1.53 (0.57, 4.09)	0.399	NC (NC, NC)	NC
	Additive	—	—	—	0.98 (0.62, 1.54)	0.922	NC (NC, NC)	NC
rs6196	Co-dominant	AA	518	2109	1.00 (ref.)	—	1.00 (ref.)	—
		GG	84	315	**2.85 (1.08, 7.52)**	**0.034***	**2.89 (1.10, 7.64)**	**0.032***
		AG	7	10	1.09 (0.84, 1.41)	0.534	1.07 (0.82, 1.39)	0.625
	Dominant	AA	518	2109	1.00 (ref.)	—	1.00 (ref.)	—
		GG+AG	91	325	1.14 (0.89, 1.47)	0.307	1.12 (0.87, 1.45)	0.369
	Recessive	GG	84	315	1.00 (ref.)	—	1.00 (ref.)	—
		AG+AA	525	2119	**2.82 (1.07, 7.44)**	**0.036***	**2.87 (1.09, 7.56)**	**0.033***
	Additive	—	—	—	1.18 (0.94, 1.49)	0.160	1.17 (0.92, 1.48)	0.194
rs258751	Co-dominant	GG	519	2112	1.00 (ref.)	—	1.00 (ref.)	—
		AA	83	314	**3.17 (1.17, 8.54)**	**0.023***	**3.21 (1.19, 8.66)**	**0.021***
		GA	7	9	1.08 (0.83, 1.40)	0.583	1.06 (0.81, 1.37)	0.678
	Dominant	GG	519	2112	1.00 (ref.)	—	1.00 (ref.)	—
		AA+GA	90	323	1.13 (0.88, 1.46)	0.330	1.12 (0.87, 1.44)	0.395
	Recessive	AA	83	314	1.00 (ref.)	—	1.00 (ref.)	—
		GA+GG	526	2121	**3.13 (1.16, 8.45)**	**0.024***	**3.19 (1.18, 8.60)**	**0.022***
	Additive	—	—	—	1.18 (0.93, 1.50)	0.163	1.17 (0.92, 1.48)	0.200
rs6194	Co-dominant	GG	519	2112	1.00 (ref.)	—	1.00 (ref.)	—
		AA	83	311	**3.56 (1.29, 9.86)**	**0.015***	**3.61 (1.30, 10.01)**	**0.013***
		GA	7	8	1.09 (0.84, 1.41)	0.535	1.07 (0.82, 1.39)	0.627
	Dominant	GG	519	2112	1.00 (ref.)	—	1.00 (ref.)	—
		AA+GA	90	319	1.15 (0.89, 1.48)	0.284	1.13 (0.88, 1.46)	0.345

 https://doi.org/10.3390/medicina62061068

	Recessive	AA	83	311	1.00 (ref.)	—	1.00 (ref.)	—
		GA+GG	526	2120	**3.52 (1.27, 9.75)**	**0.015***	**3.58 (1.29, 9.92)**	**0.014***
	Additive	—	—	—	1.20 (0.95, 1.52)	0.130	1.19 (0.93, 1.50)	0.161
rs79590198	Co-dominant	GG	460	1793	1.00 (ref.)	—	1.00 (ref.)	—
		AA	130	585	1.30 (0.75, 2.24)	0.345	1.28 (0.74, 2.21)	0.384
		GA	18	54	0.87 (0.70, 1.07)	0.192	0.87 (0.70, 1.08)	0.214
	Dominant	GG	460	1793	1.00 (ref.)	—	1.00 (ref.)	—
		AA+GA	148	639	0.90 (0.73, 1.11)	0.331	0.91 (0.74, 1.11)	0.350
	Recessive	AA	130	585	1.00 (ref.)	—	1.00 (ref.)	—
		GA+GG	478	1847	1.34 (0.78, 2.31)	0.285	1.32 (0.76, 2.27)	0.323
	Additive	—	—	—	0.95 (0.80, 1.14)	0.599	0.95 (0.80, 1.14)	0.605
rs852977	Co-dominant	AA	486	1955	1.00 (ref.)	—	1.00 (ref.)	—
		GG	111	444	1.68 (0.80, 3.53)	0.174	1.70 (0.81, 3.59)	0.161
		AG	10	24	1.01 (0.80, 1.27)	0.962	1.00 (0.79, 1.26)	0.985
	Dominant	AA	486	1955	1.00 (ref.)	—	1.00 (ref.)	—
		GG+AG	121	468	1.04 (0.83, 1.30)	0.730	1.03 (0.83, 1.29)	0.772
	Recessive	GG	111	444	1.00 (ref.)	—	1.00 (ref.)	—
		AG+AA	496	1979	1.67 (0.80, 3.52)	0.174	1.70 (0.81, 3.58)	0.160
	Additive	—	—	—	1.07 (0.87, 1.31)	0.514	1.07 (0.87, 1.31)	0.540
rs2963155	Co-dominant	AA	417	1640	1.00 (ref.)	—	1.00 (ref.)	—
		GG	165	713	1.31 (0.84, 2.05)	0.237	1.33 (0.85, 2.08)	0.215
		AG	27	81	0.91 (0.74, 1.11)	0.357	0.89 (0.73, 1.09)	0.276
	Dominant	AA	417	1640	1.00 (ref.)	—	1.00 (ref.)	—
		GG+AG	192	794	0.95 (0.79, 1.15)	0.606	0.94 (0.77, 1.14)	0.512
	Recessive	GG	165	713	1.00 (ref.)	—	1.00 (ref.)	—
		AG+AA	444	1721	1.35 (0.86, 2.10)	0.189	1.37 (0.88, 2.14)	0.163
	Additive	—	—	—	1.00 (0.85, 1.18)	0.996	0.99 (0.84, 1.17)	0.930
rs9324921	Co-dominant	CC	349	1452	1.00 (ref.)	—	1.00 (ref.)	—
		AA	228	851	1.02 (0.67, 1.55)	0.914	1.04 (0.69, 1.58)	0.841
		CA	30	122	1.11 (0.92, 1.34)	0.256	1.12 (0.93, 1.35)	0.227
	Dominant	CC	349	1452	1.00 (ref.)	—	1.00 (ref.)	—
		AA+CA	258	973	1.10 (0.92, 1.32)	0.286	1.11 (0.93, 1.33)	0.247
	Recessive	AA	228	851	1.00 (ref.)	—	1.00 (ref.)	—
		CA+CC	379	1574	0.98 (0.65, 1.48)	0.929	1.00 (0.66, 1.50)	0.994
	Additive	—	—	—	1.07 (0.92, 1.24)	0.392	1.08 (0.93, 1.25)	0.336

https://doi.org/10.3390/medicina62061068

rs11749561	Co-dominant	TT	478	1912	1.00 (ref.)	—	1.00 (ref.)	—
		CC	114	485	1.62 (0.85, 3.12)	0.145	1.65 (0.86, 3.17)	0.132
		TC	13	32	0.94 (0.75, 1.18)	0.595	0.93 (0.74, 1.17)	0.560
	Dominant	TT	478	1912	1.00 (ref.)	—	1.00 (ref.)	—
		CC+TC	127	517	0.98 (0.79, 1.22)	0.875	0.98 (0.79, 1.22)	0.846
	Recessive	CC	114	485	1.00 (ref.)	—	1.00 (ref.)	—
		TC+TT	491	1944	1.64 (0.86, 3.15)	0.134	1.67 (0.87, 3.21)	0.121
	Additive	—	—	—	1.03 (0.84, 1.25)	0.794	1.02 (0.84, 1.25)	0.809
rs7709864	Co-dominant	TT	377	1414	1.00 (ref.)	—	1.00 (ref.)	—
		CC	193	879	0.96 (0.65, 1.40)	0.825	0.94 (0.64, 1.38)	0.742
		TC	36	141	**0.82 (0.68, 1.00)**	**0.048***	**0.81 (0.67, 0.98)**	**0.033***
	Dominant	TT	377	1414	1.00 (ref.)	—	1.00 (ref.)	—
		CC+TC	229	1020	0.84 (0.70, 1.01)	0.065	**0.83 (0.69, 0.99)**	**0.043***
	Recessive	CC	193	879	1.00 (ref.)	—	1.00 (ref.)	—
		TC+TT	413	1555	1.03 (0.70, 1.50)	0.890	1.01 (0.69, 1.48)	0.958
	Additive	—	—	—	0.90 (0.77, 1.04)	0.148	0.88 (0.76, 1.03)	0.104
rs34125834	Co-dominant	GG	293	1195	1.00 (ref.)	—	1.00 (ref.)	—
		GAGA	258	1014	1.06 (0.77, 1.46)	0.733	1.08 (0.78, 1.49)	0.648
		GGA	56	216	1.04 (0.86, 1.25)	0.698	1.04 (0.86, 1.25)	0.692
	Dominant	GG	293	1195	1.00 (ref.)	—	1.00 (ref.)	—
		GAGA+GGA	314	1230	1.04 (0.87, 1.24)	0.657	1.05 (0.87, 1.25)	0.626
	Recessive	GAGA	258	1014	1.00 (ref.)	—	1.00 (ref.)	—
		GGA+GG	349	1411	1.04 (0.76, 1.41)	0.806	1.06 (0.78, 1.44)	0.717
	Additive	—	—	—	1.03 (0.90, 1.18)	0.652	1.04 (0.91, 1.19)	0.591
rs11167813	Co-dominant	TT	202	815	1.00 (ref.)	—	1.00 (ref.)	—
		GG	285	1175	1.12 (0.87, 1.44)	0.374	1.12 (0.87, 1.44)	0.383
		TG	122	439	0.98 (0.80, 1.20)	0.833	0.99 (0.81, 1.21)	0.900
	Dominant	TT	202	815	1.00 (ref.)	—	1.00 (ref.)	—
		GG+TG	407	1614	1.02 (0.84, 1.23)	0.857	1.02 (0.85, 1.24)	0.812
	Recessive	GG	285	1175	1.00 (ref.)	—	1.00 (ref.)	—
		TG+TT	324	1254	1.14 (0.91, 1.42)	0.265	1.13 (0.90, 1.41)	0.293
	Additive	—	—	—	1.05 (0.92, 1.19)	0.463	1.05 (0.92, 1.19)	0.460
rs17400549	Co-dominant	TT	516	2002	1.00 (ref.)	—	1.00 (ref.)	—
		CC	89	411	1.03 (0.34, 3.13)	0.952	1.05 (0.35, 3.18)	0.930
		TC	4	15	0.84 (0.66, 1.08)	0.170	0.83 (0.65, 1.07)	0.156

	Dominant	TT	516	2002	1.00 (ref.)	—	1.00 (ref.)	—
		CC+TC	93	426	0.85 (0.66, 1.08)	0.183	0.84 (0.66, 1.08)	0.170
	Recessive	CC	89	411	1.00 (ref.)	—	1.00 (ref.)	—
		TC+TT	520	2017	1.06 (0.35, 3.22)	0.913	1.08 (0.36, 3.27)	0.890
	Additive	—	—	—	0.86 (0.69, 1.09)	0.215	0.86 (0.68, 1.09)	0.203
rs17100326	Co-dominant	CC	530	2110	1.00 (ref.)	—	1.00 (ref.)	—
		TT	72	313	1.81 (0.67, 4.91)	0.244	1.84 (0.68, 5.00)	0.230
		CT	5	11	0.92 (0.70, 1.20)	0.528	0.91 (0.69, 1.20)	0.509
	Dominant	CC	530	2110	1.00 (ref.)	—	1.00 (ref.)	—
		TT+CT	77	324	0.95 (0.73, 1.23)	0.682	0.94 (0.72, 1.23)	0.667
	Recessive	TT	72	313	1.00 (ref.)	—	1.00 (ref.)	—
		CT+CC	535	2121	1.83 (0.67, 4.96)	0.235	1.86 (0.64, 5.39)	0.251
	Additive	—	—	—	0.98 (0.76, 1.26)	0.876	0.98 (0.76, 1.26)	0.864
rs1374001	Co-dominant	TT	499	1923	1.00 (ref.)	—	1.00 (ref.)	—
		GG	102	490	1.47 (0.66, 3.28)	0.350	1.49 (0.67, 3.34)	0.330
		TG	8	21	0.80 (0.63, 1.01)	0.066	0.80 (0.63, 1.01)	0.065
	Dominant	TT	499	1923	1.00 (ref.)	—	1.00 (ref.)	—
		GG+TG	110	511	0.83 (0.66, 1.04)	0.108	0.83 (0.66, 1.04)	0.108
	Recessive	GG	102	490	1.00 (ref.)	—	1.00 (ref.)	—
		TG+TT	507	1944	1.53 (0.68, 3.42)	0.300	1.55 (0.69, 3.53)	0.291
	Additive	—	—	—	0.87 (0.71, 1.08)	0.206	0.87 (0.70, 1.08)	0.208
rs12153243	Co-dominant	AA	376	1449	1.00 (ref.)	—	1.00 (ref.)	—
		TT	186	796	0.96 (0.58, 1.61)	0.887	0.98 (0.58, 1.64)	0.933
		AT	19	76	0.90 (0.74, 1.10)	0.294	0.89 (0.73, 1.09)	0.257
	Dominant	AA	376	1449	1.00 (ref.)	—	1.00 (ref.)	—
		TT+AT	205	872	0.91 (0.75, 1.10)	0.308	0.90 (0.74, 1.09)	0.278
	Recessive	TT	186	796	1.00 (ref.)	—	1.00 (ref.)	—
		AT+AA	395	1525	1.00 (0.60, 1.66)	0.996	1.02 (0.61, 1.70)	0.949
	Additive	—	—	—	0.93 (0.78, 1.09)	0.372	0.92 (0.78, 1.09)	0.354
rs11167820	Co-dominant	GG	237	955	1.00 (ref.)	—	1.00 (ref.)	—
		AA	292	1134	0.93 (0.70, 1.23)	0.590	0.94 (0.71, 1.25)	0.670
		GA	79	344	1.04 (0.86, 1.26)	0.706	1.04 (0.86, 1.27)	0.659
	Dominant	GG	237	955	1.00 (ref.)	—	1.00 (ref.)	—
		AA+GA	371	1478	1.01 (0.84, 1.21)	0.902	1.02 (0.85, 1.23)	0.830
	Recessive	AA	292	1134	1.00 (ref.)	—	1.00 (ref.)	—

https://doi.org/10.3390/medicina62061068

		GA+GG	316	1299	0.91 (0.70, 1.18)	0.465	0.92 (0.71, 1.19)	0.525
	Additive	—	—	—	0.98 (0.86, 1.12)	0.778	0.99 (0.87, 1.13)	0.866
rs6893954	Co-dominant	GG	197	851	1.00 (ref.)	—	1.00 (ref.)	—
		AA	320	1194	1.02 (0.77, 1.34)	0.893	1.00 (0.76, 1.32)	0.997
		GA	92	390	1.16 (0.95, 1.41)	0.147	1.15 (0.94, 1.40)	0.167
	Dominant	GG	197	851	1.00 (ref.)	—	1.00 (ref.)	—
		AA+GA	412	1584	1.12 (0.93, 1.36)	0.227	1.11 (0.92, 1.35)	0.267
	Recessive	AA	320	1194	1.00 (ref.)	—	1.00 (ref.)	—
		GA+GG	289	1241	0.93 (0.73, 1.19)	0.582	0.92 (0.72, 1.18)	0.510
	Additive	—	—	—	1.04 (0.91, 1.18)	0.585	1.03 (0.90, 1.17)	0.675
rs4912927	Co-dominant	AA	152	592	1.00 (ref.)	—	1.00 (ref.)	—
		GG	320	1316	1.01 (0.78, 1.30)	0.967	1.00 (0.77, 1.29)	0.980
		AG	134	519	0.95 (0.76, 1.18)	0.621	0.94 (0.76, 1.17)	0.569
	Dominant	AA	152	592	1.00 (ref.)	—	1.00 (ref.)	—
		GG+AG	454	1835	0.96 (0.78, 1.18)	0.723	0.96 (0.78, 1.17)	0.663
	Recessive	GG	320	1316	1.00 (ref.)	—	1.00 (ref.)	—
		AG+AA	286	1111	1.04 (0.84, 1.29)	0.696	1.04 (0.84, 1.29)	0.718
	Additive	—	—	—	1.00 (0.88, 1.14)	0.990	1.00 (0.87, 1.14)	0.955
rs17100411	Co-dominant	AA	356	1394	1.00 (ref.)	—	1.00 (ref.)	—
		CC	218	917	1.18 (0.80, 1.75)	0.409	1.18 (0.80, 1.76)	0.404
		AC	35	116	0.93 (0.77, 1.12)	0.455	0.93 (0.77, 1.12)	0.458
	Dominant	AA	356	1394	1.00 (ref.)	—	1.00 (ref.)	—
		CC+AC	253	1033	0.96 (0.80, 1.15)	0.649	0.96 (0.80, 1.15)	0.654
	Recessive	CC	218	917	1.00 (ref.)	—	1.00 (ref.)	—
		AC+AA	391	1510	1.21 (0.82, 1.79)	0.327	1.22 (0.82, 1.80)	0.323
	Additive	—	—	—	1.00 (0.86, 1.16)	0.985	1.00 (0.86, 1.16)	0.992
rs153516	Co-dominant	AA	143	618	1.00 (ref.)	—	1.00 (ref.)	—
		GG	329	1204	0.96 (0.74, 1.25)	0.766	0.96 (0.74, 1.25)	0.762
		AG	135	607	1.18 (0.95, 1.47)	0.137	1.18 (0.95, 1.47)	0.133
	Dominant	AA	143	618	1.00 (ref.)	—	1.00 (ref.)	—
		GG+AG	464	1811	1.11 (0.90, 1.36)	0.338	1.11 (0.90, 1.37)	0.334
	Recessive	GG	329	1204	1.00 (ref.)	—	1.00 (ref.)	—
		AG+AA	278	1225	0.86 (0.69, 1.06)	0.159	0.86 (0.69, 1.06)	0.154
	Additive	—	—	—	0.98 (0.87, 1.12)	0.786	0.98 (0.87, 1.12)	0.783
rs1582888	Co-dominant	AA	482	1915	1.00 (ref.)	—	1.00 (ref.)	—

		GG	118	489	1.19 (0.56, 2.53)	0.647	1.07 (0.49, 2.31)	0.867
		AG	9	30	0.96 (0.77, 1.20)	0.713	0.97 (0.78, 1.22)	0.806
	Dominant	AA	482	1915	1.00 (ref.)	—	1.00 (ref.)	—
		GG+AG	127	519	0.97 (0.78, 1.21)	0.799	0.98 (0.79, 1.22)	0.843
	Recessive	GG	118	489	1.00 (ref.)	—	1.00 (ref.)	—
		AG+AA	491	1945	1.20 (0.59, 2.43)	0.609	1.07 (0.50, 2.32)	0.856
	Additive	—	—	—	0.99 (0.81, 1.21)	0.912	0.99 (0.81, 1.20)	0.892
rs17404863	Co-dominant	TT	378	1480	1.00 (ref.)	—	1.00 (ref.)	—
		CC	207	835	0.75 (0.47, 1.20)	0.228	0.76 (0.48, 1.22)	0.258
		TC	22	115	0.97 (0.80, 1.17)	0.758	0.97 (0.80, 1.18)	0.766
	Dominant	TT	378	1480	1.00 (ref.)	—	1.00 (ref.)	—
		CC+TC	229	950	0.94 (0.79, 1.13)	0.536	0.95 (0.79, 1.14)	0.557
	Recessive	CC	207	835	1.00 (ref.)	—	1.00 (ref.)	—
		TC+TT	400	1595	0.76 (0.48, 1.21)	0.241	0.77 (0.48, 1.23)	0.271
	Additive	—	—	—	0.93 (0.79, 1.08)	0.346	0.93 (0.80, 1.09)	0.374
rs247623	Co-dominant	TT	507	1989	1.00 (ref.)	—	1.00 (ref.)	—
		GG	96	414	0.58 (0.20, 1.67)	0.313	0.59 (0.21, 1.70)	0.328
		TG	4	27	0.91 (0.71, 1.16)	0.444	0.91 (0.71, 1.16)	0.453
	Dominant	TT	507	1989	1.00 (ref.)	—	1.00 (ref.)	—
		GG+TG	100	441	0.89 (0.70, 1.13)	0.335	0.89 (0.70, 1.13)	0.346
	Recessive	GG	96	414	1.00 (ref.)	—	1.00 (ref.)	—
		TG+TT	511	2016	0.59 (0.21, 1.69)	0.327	0.60 (0.21, 1.72)	0.342
	Additive	—	—	—	0.88 (0.71, 1.10)	0.261	0.88 (0.71, 1.10)	0.273
rs12659355	Co-dominant	TT	287	1221	1.00 (ref.)	—	1.00 (ref.)	—
		CC	273	1014	1.11 (0.78, 1.58)	0.551	1.12 (0.79, 1.60)	0.522
		TC	45	172	1.15 (0.95, 1.38)	0.151	1.15 (0.95, 1.39)	0.140
	Dominant	TT	287	1221	1.00 (ref.)	—	1.00 (ref.)	—
		CC+TC	318	1186	1.14 (0.95, 1.36)	0.148	1.15 (0.96, 1.37)	0.135
	Recessive	CC	273	1014	1.00 (ref.)	—	1.00 (ref.)	—
		TC+TT	332	1393	1.04 (0.74, 1.47)	0.804	1.05 (0.75, 1.48)	0.776
	Additive	—	—	—	1.10 (0.95, 1.26)	0.207	1.10 (0.95, 1.27)	0.188
rs11738413	Co-dominant	TT	280	1080	1.00 (ref.)	—	1.00 (ref.)	—
		CC	244	1040	1.03 (0.78, 1.36)	0.833	1.03 (0.78, 1.36)	0.827
		TC	82	307	0.90 (0.75, 1.10)	0.307	0.91 (0.75, 1.11)	0.353
	Dominant	TT	280	1080	1.00 (ref.)	—	1.00 (ref.)	—

https://doi.org/10.3390/medicina62061068

		CC+TC	326	1347	0.93 (0.78, 1.12)	0.450	0.94 (0.79, 1.12)	0.498
	Recessive	CC	244	1040	1.00 (ref.)	—	1.00 (ref.)	—
		TC+TT	362	1387	1.08 (0.83, 1.40)	0.561	1.08 (0.83, 1.40)	0.576
	Additive	—	—	—	0.98 (0.86, 1.12)	0.792	0.99 (0.87, 1.12)	0.828
rs56318527	Co-dominant	TT	227	896	1.00 (ref.)	—	1.00 (ref.)	—
		CC	223	980	1.16 (0.87, 1.55)	0.304	1.16 (0.87, 1.55)	0.306
		TC	81	275	0.90 (0.73, 1.10)	0.306	0.91 (0.74, 1.12)	0.361
	Dominant	TT	227	896	1.00 (ref.)	—	1.00 (ref.)	—
		CC+TC	304	1255	0.96 (0.79, 1.16)	0.647	0.96 (0.80, 1.17)	0.711
	Recessive	CC	223	980	1.00 (ref.)	—	1.00 (ref.)	—
		TC+TT	308	1171	1.23 (0.94, 1.61)	0.134	1.22 (0.93, 1.60)	0.146
	Additive	—	—	—	1.03 (0.90, 1.18)	0.679	1.03 (0.90, 1.19)	0.650
rs10063590	Co-dominant	TT	252	940	1.00 (ref.)	—	1.00 (ref.)	—
		GG	270	1138	0.92 (0.70, 1.21)	0.546	0.93 (0.70, 1.22)	0.588
		TG	85	345	0.89 (0.73, 1.07)	0.213	0.88 (0.72, 1.06)	0.183
	Dominant	TT	252	940	1.00 (ref.)	—	1.00 (ref.)	—
		GG+TG	355	1483	0.89 (0.75, 1.07)	0.219	0.89 (0.74, 1.07)	0.203
	Recessive	GG	270	1138	1.00 (ref.)	—	1.00 (ref.)	—
		TG+TT	337	1285	0.98 (0.76, 1.27)	0.882	0.99 (0.77, 1.28)	0.960
	Additive	—	—	—	0.94 (0.82, 1.07)	0.343	0.94 (0.82, 1.07)	0.352
rs1213706	Co-dominant	AA	408	1607	1.00 (ref.)	—	1.00 (ref.)	—
		GG	178	744	0.97 (0.59, 1.60)	0.913	0.99 (0.60, 1.63)	0.968
		AG	20	81	0.94 (0.77, 1.15)	0.553	0.94 (0.77, 1.15)	0.558
	Dominant	AA	408	1607	1.00 (ref.)	—	1.00 (ref.)	—
		GG+AG	198	825	0.95 (0.78, 1.14)	0.560	0.95 (0.78, 1.15)	0.577
	Recessive	GG	178	744	1.00 (ref.)	—	1.00 (ref.)	—
		AG+AA	428	1688	0.99 (0.60, 1.62)	0.970	1.01 (0.61, 1.66)	0.975
	Additive	—	—	—	0.96 (0.81, 1.13)	0.607	0.96 (0.82, 1.13)	0.637
rs2910256	Co-dominant	GG	147	650	1.00 (ref.)	—	1.00 (ref.)	—
		AA	304	1188	1.17 (0.91, 1.51)	0.218	1.16 (0.90, 1.50)	0.243
		GA	155	585	1.13 (0.91, 1.41)	0.269	1.12 (0.90, 1.40)	0.309
	Dominant	GG	147	650	1.00 (ref.)	—	1.00 (ref.)	—
		AA+GA	459	1773	1.14 (0.93, 1.41)	0.198	1.13 (0.92, 1.39)	0.231
	Recessive	AA	304	1188	1.00 (ref.)	—	1.00 (ref.)	—
		GA+GG	302	1235	1.08 (0.88, 1.33)	0.463	1.08 (0.88, 1.32)	0.473

https://doi.org/10.3390/medicina62061068

	Additive	—	—	—	1.08 (0.95, 1.23)	0.216	1.08 (0.95, 1.22)	0.241
rs1427864	Co-dominant	AA	271	1042	1.00 (ref.)	—	1.00 (ref.)	—
		CC	267	1109	1.03 (0.76, 1.39)	0.865	1.05 (0.78, 1.42)	0.748
		AC	67	251	0.93 (0.77, 1.12)	0.423	0.95 (0.78, 1.15)	0.594
	Dominant	AA	271	1042	1.00 (ref.)	—	1.00 (ref.)	—
		CC+AC	334	1360	0.94 (0.79, 1.13)	0.531	0.97 (0.81, 1.16)	0.726
	Recessive	CC	267	1109	1.00 (ref.)	—	1.00 (ref.)	—
		AC+AA	338	1293	1.07 (0.80, 1.42)	0.655	1.08 (0.81, 1.44)	0.604
	Additive	—	—	—	0.98 (0.86, 1.12)	0.792	1.00 (0.87, 1.14)	0.984
rs890710	Co-dominant	CC	284	1270	1.00 (ref.)	—	1.00 (ref.)	—
		TT	280	971	1.05 (0.75, 1.49)	0.767	1.07 (0.75, 1.51)	0.715
		CT	45	191	**1.29 (1.07, 1.55)**	**0.007****	**1.28 (1.06, 1.54)**	**0.010***
	Dominant	CC	284	1270	1.00 (ref.)	—	1.00 (ref.)	—
		TT+CT	325	1162	**1.25 (1.05, 1.49)**	**0.013***	**1.24 (1.04, 1.48)**	**0.018***
	Recessive	TT	280	971	1.00 (ref.)	—	1.00 (ref.)	—
		CT+CC	329	1461	0.94 (0.67, 1.31)	0.699	0.95 (0.68, 1.33)	0.779
	Additive	—	—	—	1.13 (0.99, 1.30)	0.074	1.13 (0.99, 1.30)	0.080
rs10061924	Co-dominant	AA	230	938	1.00 (ref.)	—	1.00 (ref.)	—
		GG	293	1132	0.97 (0.74, 1.28)	0.853	0.99 (0.75, 1.30)	0.933
		AG	86	360	1.06 (0.87, 1.28)	0.583	1.07 (0.88, 1.30)	0.502
	Dominant	AA	230	938	1.00 (ref.)	—	1.00 (ref.)	—
		GG+AG	379	1492	1.04 (0.86, 1.24)	0.705	1.05 (0.87, 1.26)	0.608
	Recessive	GG	293	1132	1.00 (ref.)	—	1.00 (ref.)	—
		AG+AA	316	1298	0.95 (0.73, 1.22)	0.666	0.95 (0.74, 1.23)	0.708
	Additive	—	—	—	1.00 (0.88, 1.14)	0.964	1.01 (0.89, 1.15)	0.865
rs12717905	Co-dominant	GG	163	628	1.00 (ref.)	—	1.00 (ref.)	—
		AA	309	1211	0.89 (0.69, 1.15)	0.387	0.89 (0.69, 1.15)	0.373
		GA	135	582	0.98 (0.79, 1.22)	0.875	0.97 (0.79, 1.21)	0.806
	Dominant	GG	163	628	1.00 (ref.)	—	1.00 (ref.)	—
		AA+GA	444	1793	0.95 (0.78, 1.17)	0.647	0.95 (0.77, 1.16)	0.594
	Recessive	AA	309	1211	1.00 (ref.)	—	1.00 (ref.)	—
		GA+GG	298	1210	0.90 (0.73, 1.12)	0.351	0.91 (0.73, 1.12)	0.366
	Additive	—	—	—	0.95 (0.83, 1.07)	0.397	0.94 (0.83, 1.07)	0.380
rs17413459	Co-dominant	GG	463	1947	1.00 (ref.)	—	1.00 (ref.)	—
		AA	142	460	0.60 (0.21, 1.72)	0.343	0.61 (0.21, 1.75)	0.356

https://doi.org/10.3390/medicina62061068

		GA	4	28	**1.30 (1.05, 1.61)**	**0.017***	**1.28 (1.03, 1.58)**	**0.027***
	Dominant	GG	463	1947	1.00 (ref.)	—	1.00 (ref.)	—
		AA+GA	146	488	**1.26 (1.02, 1.55)**	**0.033***	**1.24 (1.00, 1.53)**	**0.049***
	Recessive	AA	142	460	1.00 (ref.)	—	1.00 (ref.)	—
		GA+GG	467	1975	0.57 (0.20, 1.63)	0.292	0.58 (0.20, 1.66)	0.307
	Additive	—	—	—	1.19 (0.98, 1.44)	0.084	1.17 (0.96, 1.43)	0.112
rs6862531	Co-dominant	GG	461	1944	1.00 (ref.)	—	1.00 (ref.)	—
		AA	142	461	0.60 (0.21, 1.73)	0.345	0.61 (0.21, 1.75)	0.359
		GA	4	28	**1.30 (1.05, 1.61)**	**0.016***	**1.28 (1.03, 1.58)**	**0.026***
	Dominant	GG	461	1944	1.00 (ref.)	—	1.00 (ref.)	—
		AA+GA	146	489	**1.26 (1.02, 1.55)**	**0.032***	**1.24 (1.00, 1.53)**	**0.048***
	Recessive	AA	142	461	1.00 (ref.)	—	1.00 (ref.)	—
		GA+GG	465	1972	0.57 (0.20, 1.63)	0.294	0.58 (0.20, 1.66)	0.309
	Additive	—	—	—	1.19 (0.98, 1.45)	0.082	1.17 (0.96, 1.43)	0.110
rs74567155	Co-dominant	CCTTCCTT	278	1116	1.00 (ref.)	—	1.00 (ref.)	—
		CC	275	1039	0.78 (0.56, 1.08)	0.129	0.78 (0.57, 1.08)	0.136
		CCTTC	53	273	1.06 (0.88, 1.28)	0.525	1.05 (0.87, 1.27)	0.607
	Dominant	CCTTCCTT	278	1116	1.00 (ref.)	—	1.00 (ref.)	—
		CC+CCTTC	328	1312	1.00 (0.84, 1.20)	0.969	0.99 (0.83, 1.19)	0.955
	Recessive	CC	275	1039	1.00 (ref.)	—	1.00 (ref.)	—
		CCTTC+CCTTCCTT	331	1389	0.76 (0.56, 1.03)	0.076	0.76 (0.56, 1.04)	0.087
	Additive	—	—	—	0.95 (0.83, 1.08)	0.426	0.94 (0.82, 1.08)	0.401
rs116972667	Co-dominant	GG	543	2199	1.00 (ref.)	—	1.00 (ref.)	—
		AA	S	S	0.51 (0.09, 2.76)	0.431	0.51 (0.09, 2.80)	0.442
		GA	S	S	1.19 (0.89, 1.59)	0.253	1.19 (0.89, 1.59)	0.251
	Dominant	GG	543	2199	1.00 (ref.)	—	1.00 (ref.)	—
		AA+GA	66	230	1.16 (0.87, 1.55)	0.307	1.16 (0.87, 1.56)	0.306
	Recessive	AA	S	S	1.00 (ref.)	—	1.00 (ref.)	—
		GA+GG	S	S	0.50 (0.09, 2.71)	0.420	0.51 (0.06, 4.05)	0.521
	Additive	—	—	—	1.13 (0.86, 1.49)	0.392	1.13 (0.86, 1.49)	0.387
rs1387791	Co-dominant	TT	312	1299	1.00 (ref.)	—	1.00 (ref.)	—
		CC	249	947	1.05 (0.74, 1.49)	0.773	1.06 (0.75, 1.50)	0.737
		TC	46	182	1.09 (0.91, 1.32)	0.341	1.10 (0.91, 1.32)	0.337
	Dominant	TT	312	1299	1.00 (ref.)	—	1.00 (ref.)	—
		CC+TC	295	1129	1.09 (0.91, 1.30)	0.354	1.09 (0.91, 1.30)	0.343

https://doi.org/10.3390/medicina62061068

	Recessive	CC	249	947	1.00 (ref.)	—	1.00 (ref.)	—
		TC+TT	358	1481	1.01 (0.72, 1.42)	0.945	1.02 (0.73, 1.43)	0.910
	Additive	—	—	—	1.06 (0.92, 1.21)	0.446	1.06 (0.92, 1.22)	0.424
rs10515524	Co-dominant	GG	175	748	1.00 (ref.)	—	1.00 (ref.)	—
		AA	310	1167	1.01 (0.78, 1.30)	0.965	1.01 (0.78, 1.31)	0.922
		GA	120	510	1.14 (0.92, 1.40)	0.229	1.14 (0.92, 1.40)	0.223
	Dominant	GG	175	748	1.00 (ref.)	—	1.00 (ref.)	—
		AA+GA	430	1677	1.10 (0.90, 1.33)	0.358	1.10 (0.90, 1.34)	0.341
	Recessive	AA	310	1167	1.00 (ref.)	—	1.00 (ref.)	—
		GA+GG	295	1258	0.93 (0.74, 1.16)	0.517	0.93 (0.75, 1.17)	0.553
	Additive	—	—	—	1.01 (0.89, 1.15)	0.822	1.02 (0.90, 1.16)	0.780
rs3805462	Co-dominant	TT	234	932	1.00 (ref.)	—	1.00 (ref.)	—
		GG	291	1128	0.91 (0.69, 1.20)	0.499	0.90 (0.68, 1.19)	0.473
		TG	84	368	1.03 (0.85, 1.25)	0.783	1.03 (0.85, 1.25)	0.763
	Dominant	TT	234	932	1.00 (ref.)	—	1.00 (ref.)	—
		GG+TG	375	1496	1.00 (0.83, 1.20)	0.986	1.00 (0.83, 1.20)	0.991
	Recessive	GG	291	1128	1.00 (ref.)	—	1.00 (ref.)	—
		TG+TT	318	1300	0.90 (0.69, 1.16)	0.397	0.89 (0.69, 1.15)	0.368
	Additive	—	—	—	0.97 (0.85, 1.10)	0.654	0.97 (0.85, 1.10)	0.637
rs12519988	Co-dominant	CC	419	1636	1.00 (ref.)	—	1.00 (ref.)	—
		TT	177	726	0.54 (0.27, 1.09)	0.087	0.53 (0.26, 1.08)	0.079
		CT	9	65	0.95 (0.78, 1.16)	0.623	0.96 (0.79, 1.17)	0.670
	Dominant	CC	419	1636	1.00 (ref.)	—	1.00 (ref.)	—
		TT+CT	186	791	0.92 (0.76, 1.11)	0.384	0.92 (0.76, 1.12)	0.412
	Recessive	TT	177	726	1.00 (ref.)	—	1.00 (ref.)	—
		CT+CC	428	1701	0.55 (0.27, 1.11)	0.094	0.54 (0.26, 1.09)	0.084
	Additive	—	—	—	0.89 (0.75, 1.06)	0.203	0.90 (0.75, 1.07)	0.213
rs13165784	Co-dominant	CC	456	1882	1.00 (ref.)	—	1.00 (ref.)	—
		TT	142	512	1.06 (0.53, 2.09)	0.871	1.08 (0.55, 2.14)	0.825
		CT	10	39	1.14 (0.93, 1.42)	0.212	1.16 (0.94, 1.43)	0.173
	Dominant	CC	456	1882	1.00 (ref.)	—	1.00 (ref.)	—
		TT+CT	152	551	1.14 (0.93, 1.40)	0.217	1.15 (0.94, 1.42)	0.176
	Recessive	TT	142	512	1.00 (ref.)	—	1.00 (ref.)	—
		CT+CC	466	1921	1.03 (0.52, 2.03)	0.940	1.04 (0.52, 2.08)	0.902
	Additive	—	—	—	1.11 (0.92, 1.34)	0.259	1.13 (0.93, 1.36)	0.210

rs12518693	Co-dominant	TT	398	1530	1.00 (ref.)	—	1.00 (ref.)	—
		GG	197	815	0.58 (0.32, 1.05)	0.073	0.58 (0.32, 1.05)	0.070
		TG	13	86	0.93 (0.77, 1.12)	0.451	0.94 (0.77, 1.13)	0.507
	Dominant	TT	398	1530	1.00 (ref.)	—	1.00 (ref.)	—
		GG+TG	210	901	0.90 (0.74, 1.08)	0.248	0.90 (0.75, 1.09)	0.281
	Recessive	GG	197	815	1.00 (ref.)	—	1.00 (ref.)	—
		TG+TT	411	1616	0.60 (0.33, 1.07)	0.085	0.59 (0.33, 1.07)	0.080
	Additive	—	—	—	0.88 (0.74, 1.03)	0.117	0.88 (0.75, 1.04)	0.132
rs7717240	Co-dominant	CC	445	1764	1.00 (ref.)	—	1.00 (ref.)	—
		TT	147	605	1.08 (0.61, 1.93)	0.792	1.10 (0.62, 1.97)	0.740
		CT	15	55	0.96 (0.78, 1.19)	0.724	0.97 (0.79, 1.20)	0.800
	Dominant	CC	445	1764	1.00 (ref.)	—	1.00 (ref.)	—
		TT+CT	162	660	0.97 (0.80, 1.19)	0.789	0.98 (0.80, 1.20)	0.876
	Recessive	TT	147	605	1.00 (ref.)	—	1.00 (ref.)	—
		CT+CC	460	1819	1.09 (0.61, 1.95)	0.767	1.11 (0.62, 1.98)	0.722
	Additive	—	—	—	0.99 (0.83, 1.18)	0.883	1.00 (0.83, 1.19)	0.975
rs28593206	Co-dominant	GG	168	789	1.00 (ref.)	—	1.00 (ref.)	—
		AA	313	1193	**1.33 (1.02, 1.72)**	**0.033***	**1.34 (1.03, 1.74)**	**0.027***
		GA	124	439	**1.23 (1.00, 1.52)**	**0.049***	**1.24 (1.00, 1.52)**	**0.047***
	Dominant	GG	168	789	1.00 (ref.)	—	1.00 (ref.)	—
		AA+GA	437	1632	**1.26 (1.03, 1.53)**	**0.023***	**1.26 (1.04, 1.54)**	**0.020***
	Recessive	AA	313	1193	1.00 (ref.)	—	1.00 (ref.)	—
		GA+GG	292	1228	1.16 (0.93, 1.45)	0.182	1.18 (0.94, 1.47)	0.156
	Additive	—	—	—	**1.16 (1.02, 1.32)**	**0.023***	**1.17 (1.03, 1.32)**	**0.019***
rs315180	Co-dominant	GG	467	1848	1.00 (ref.)	—	1.00 (ref.)	—
		AA	128	547	1.39 (0.73, 2.64)	0.313	1.42 (0.75, 2.69)	0.286
		GA	13	37	0.93 (0.74, 1.15)	0.489	0.93 (0.75, 1.16)	0.536
	Dominant	GG	467	1848	1.00 (ref.)	—	1.00 (ref.)	—
		AA+GA	141	584	0.96 (0.77, 1.18)	0.670	0.96 (0.78, 1.19)	0.732
	Recessive	AA	128	547	1.00 (ref.)	—	1.00 (ref.)	—
		GA+GG	480	1885	1.41 (0.75, 2.68)	0.287	1.44 (0.76, 2.72)	0.264
	Additive	—	—	—	0.99 (0.82, 1.20)	0.923	1.00 (0.83, 1.21)	0.995
rs315199	Co-dominant	GG	206	945	1.00 (ref.)	—	1.00 (ref.)	—
		AA	296	1140	**1.40 (1.07, 1.83)**	**0.013***	**1.42 (1.09, 1.85)**	**0.010***
		GA	105	344	1.19 (0.98, 1.45)	0.083	1.19 (0.98, 1.45)	0.087

https://doi.org/10.3390/medicina62061068

	Dominant	GG	206	945	1.00 (ref.)	—	1.00 (ref.)	—
		AA+GA	401	1484	**1.24 (1.03, 1.49)**	**0.024***	**1.24 (1.03, 1.50)**	**0.023***
	Recessive	AA	296	1140	1.00 (ref.)	—	1.00 (ref.)	—
		GA+GG	311	1289	1.27 (1.00, 1.61)	0.052	**1.29 (1.01, 1.64)**	**0.039***
	Additive	—	—	—	**1.18 (1.04, 1.35)**	**0.010***	**1.19 (1.05, 1.35)**	**0.008****
rs12658672	Co-dominant	GG	366	1531	1.00 (ref.)	—	1.00 (ref.)	—
		AA	201	786	**1.58 (1.08, 2.33)**	**0.019***	**1.62 (1.10, 2.38)**	**0.015***
		GA	39	103	1.07 (0.88, 1.30)	0.492	1.08 (0.89, 1.31)	0.442
	Dominant	GG	366	1531	1.00 (ref.)	—	1.00 (ref.)	—
		AA+GA	240	889	1.13 (0.94, 1.36)	0.191	1.14 (0.95, 1.37)	0.159
	Recessive	AA	201	786	1.00 (ref.)	—	1.00 (ref.)	—
		GA+GG	405	1634	**1.55 (1.06, 2.26)**	**0.024***	**1.57 (1.08, 2.30)**	**0.019***
	Additive	—	—	—	1.16 (1.00, 1.34)	0.056	**1.17 (1.01, 1.36)**	**0.042***

[1] Crude odds ratio.

[2] Adjusted odds ratio (adjusted for age and sex).

*$p < 0.05$, **$p < 0.01$, and ***$p < 0.001$.

NC: Not calculated because the statistical model failed to converge due to insufficient subgroup sample size after adjustment.

S: Suppressed to protect participant confidentiality because the cell count was less than three.

—: Not applicable.

No formal correction for multiple testing was applied because this study was designed as an exploratory, hypothesis-generating candidate-gene analysis. Therefore, the reported associations should be interpreted as nominal findings requiring independent validation.

Genotype counts represent the number of participants with valid genotype calls for each SNP and inheritance model. Because locus-specific quality-control filtering was applied, the effective sample size varied slightly across SNPs.

NC: Not calculated (the adjusted odds ratio could not be estimated as the statistical model failed to converge due to an insufficient number of subjects in certain subgroups required for the adjustment).

S: Suppressed to protect participant confidentiality due to a cell count of less than three.

—: Not applicable.

No formal correction for multiple testing was applied because this study was designed as an exploratory, hypothesis-generating candidate-gene analysis focusing on biologically plausible variants within NR3C1. Applying overly stringent corrections in this context may increase the risk of type II error and obscure potentially relevant genetic signals requiring further validation.

Genotype counts represent the number of participants with valid genotype calls for each SNP and inheritance model. Because locus-specific quality-control filtering was applied, the effective sample size varied slightly across SNPs.

https://doi.org/10.3390/medicina62061068
